# Vitamin D Deficiency Causes Defective Resistance to *Aspergillus fumigatus* in Mice via Aggravated and Sustained Inflammation

**DOI:** 10.1371/journal.pone.0099805

**Published:** 2014-06-13

**Authors:** Pei Li, Xiaoyong Xu, Ehong Cao, Bo Yu, Wanchun Li, Ming Fan, Mei Huang, Lining Shi, Rong Zeng, Xin Su, Yi Shi

**Affiliations:** 1 Department of Respiratory and Critical Care Medicine, Jinling Hospital, Nanjing University School of Medicine, Nanjing Jiangsu Province, People's Republic of China; 2 Department of Pathology, Jinling Hospital, Nanjing University School of Medicine, Nanjing Jiangsu Province, People's Republic of China; 3 Department of Clinical Microbiology, Jinling Hospital, Nanjing University School of Medicine, Nanjing Jiangsu Province, People's Republic of China; 4 Department of Clinical Laboratory, Jinling Hospital, Nanjing University School of Medicine, Nanjing Jiangsu Province, People's Republic of China; Louisiana State University, United States of America

## Abstract

**Background:**

Vitamin D plays an important role in pulmonary resistance and immunity, and its deficiency has been linked to various respiratory infections. Little is known about the effect of vitamin D deficiency on host pulmonary defense to *Aspergillus fumigatus* (*A. fumigatus*).

**Methods:**

Mice raised on vitamin D sufficient or deficient diets were infected intratracheally with *A. fumigatus* conidia. Mortality, fungal growth, weight loss and lung histology were monitored. Alveolar macrophages (AMs) were stimulated with *A. fumigatus* conidia *in vitro*. The kinetics of pro-inflammatory cytokines (TNF-α, IL-1β and IL-6), chemokines (CXCL1, CCL3), and pattern recognition receptors (Toll-like receptor [TLR] 2, TLR 4 and dectin-1) expression in the lungs and AMs were measured.

**Results:**

Upon *A. fumigatus* infection, vitamin D deficient mice showed higher mortality, greater fungal load, and more weight loss than its sufficient counterparts. Vitamin D deficient mice demonstrated aggravated and prolonged histological evidence of lung inflammation as well as enhanced BAL cell counts, dominated by neutrophils after *A. fumigatus* inoculation. Increased basal levels of pro-inflammatory cytokines in the lungs and AMs from naïve vitamin D deficient mice were observed. Upon *A. fumigatus* exposure, vitamin D deficiency led to enhanced and sustained expression of TNF-α, IL-1β, IL-6, CXCL1 and CCL3 both *in vivo* and *in vitro*. Up-regulation of TLR2, TLR4 and dectin-1was observed in the lungs and AMs from vitamin D deficient mice both at baseline and after *A. fumigatus* exposure.

**Conclusions:**

Vitamin D deficiency causes defective pulmonary resistance to *A. fumigatus* in mice, possibly by the enhanced basal expression of pattern recognition receptors and pro-inflammatory cytokines, which induced excessive inflammatory response in response to *A. fumigatus* challenge.

## Introduction

It is increasingly recognized that vitamin D plays an important role in pulmonary defense, immunity, and inflammatory processes [1], [2]. The active vitamin D generating enzyme, 1α-hydroxylase, is expressed by the airway epithelium[3], alveolar macrophages (AMs)[4] and dendritic cells[5], indicating that active vitamin D can be produced locally within the lungs, that it acts in an autocrine or paracrine fashion, and is responsible for many immunomodulatory actions. Immune effects of vitamin D include increased secretion of the antimicrobial peptide cathelicidin, decreased chemokine production, inhibition of dendritic cell activation, and alteration of T-cell activation [6]. These cellular effects are important for host responses against infection and impaired vitamin D status would compromise the immune activity of vitamin D.

Vitamin D deficiency and insufficiency is a global issue which has significant implications for health [7], [8]. Multiple studies have identified a correlation between vitamin D deficiency and various respiratory infections, especially increased risk of tuberculosis, influenza and viral respiratory tract infections [9], [10], [11], [12]. *A. fumigatus* remains the most common Aspergillus species that cause respiratory infections in humans, in particular in the immunocompromised patient population [13]. However, little has been done to assess the impact of impaired vitamin D status on the host control of pulmonary *A. fumigatus* infection. Vitamin D deficiency has been proposed as a risk factor for Allergic Bronchopulmonary Aspergillosis (ABPA) in cystic fibrosis (CF) patients[14]. Based on the fact that local synthesis of active vitamin D in lung can act to support immunomodulation at this barrier site, we hypothesized that vitamin D deficiency may compromise pulmonary resistance to *A. fumigatus.* In the current study, we sought to test this hypothesis by inducing pulmonary *A. fumigatus* infection in mice raised on vitamin D sufficient (VitD+) or vitamin D deficient (VitD-) diets.

## Materials and Methods

### Animals

This study was carried out in strict accordance with the recommendations in the Guide for the Care and Use of Laboratory Animals of the National Institutes of Health. All mouse experiments were approved by the Animal Care and Use Committee at Nanjing University and Jingling Hospital under university-approved standards. Three-week old female C57BL/6J mice (Institute of Laboratory Animal Sciences, the Chinese Academy of Medical Sciences) were housed in groups of five together on hardwood chip bedding in micro-isolator cages in specific pathogen-free enviroment within small animal care facilities, at a room temperature of 22.5±1°C, and a relative humidity of 52±5%. Mice were exposed to a 12 hr light/dark cycle and had ad libitum access to the assigned diet and tap water. Cage maintenance was performed twice weekly, and the animals were handled by the same individuals at all times. Mice were weighed weekly and monitored for changes in health status. After one week acclimatization, the mice were randomized to VitD- diet (AIN-93G/No vitamin D) or VitD+ diet (AIN-93G) for at least eight weeks. The vitamin D replete diet contained 1000 IU vitamin D3/kg of food. All diets were procured from Research Diets, Inc. (New Brunswick, NJ). Every two weeks, three mice from each group were bled from the retro-orbital venous plexus. The blood was centrifuged at 1,000–1,500 rpm for 10 min and the serum collected for 25-OH VitD3 (25-(OH)D3) analysis. Serum 25-(OH)D3 levels were measured by ELISA kit (IDS) to confirm vitamin D deficiency. Ketamine-xylazine anesthesia was used for retro-orbital venous plexus puncture. Under ketamine-xylazine anesthesia, animals were sacrificed by cervical dislocation for each experiment. All efforts were made to minimize animal suffering.

### Preparation of *A. fumigatus*

The strain of *A. fumigatus* was obtained from a patient with a fatal case of pulmonary aspergillosis at the Department of Respiratory and Critical Care Medicine, Jinling Hospital, Nanjing University School of Medicine. Conidia were harvested by washing a 7 day-old slant culture on Sabouraud dextrose agar with PBS supplemented with 0.1% Tween 20. The suspension was filtered through a 40 µm cell strainer (Falcon) to separate conidia from the contaminating mycelium and the absence of mycelium in the filtrate was verified microscopically.

### In vivo *A. fumigatus* infection, survival and tissue burden assessment, and histology

Mice were lightly anesthetized with ketamine-xylazine and administered intratracheally with 2–7×10^7^ per 50 µl viable conidia while being held in a vertical position. Afterword, mice were placed on their backs during recovery from anesthesia. After inoculation, all animals fully recovered within 1–2 hrs and were normal in appearance until signs of disease became apparent 24–30 hrs after infection. Survival, clinical appearance and body weight were monitored daily for each group. For survival studies, mice were monitored every 6 hr post-*A. fumigatus* challenge. Mice were observed on a regular basis during the day and were weighed each morning. Body temperature was taken in the morning and evening with a digital thermometer inserted into the vagina. Time of death or premature euthanasia was recorded, and deaths that occurred at night were assigned a time of death midway between the last evening observation and the first morning observation. Criteria for premature euthanasia were labored breathing, a 25% weight loss, and severe hypothermia (body temperature <32°C). Fungal burden in lungs was determined by a quantitative colony forming units (CFUs) assay. At selected time points post infection (p.i.) lungs were aseptically removed and their wet masses determined using a precision balance (±0.01 g). Tissue specimens were homogenized using a mechanical homogenizer for 1 min at a 1∶5 dilution (w/v) in sterile PBS. Primary homogenate dilutions were quantitatively cultured by serial dilution, plated in triplicate on SDA plates, incubated at 37°C for 24–48 hrs, and the number of CFUs per gram of tissue was enumerated. Results were expressed as log_10_CFU per lung. Sections of formalin fixed lung were stained with hematoxylin and eosin (HE) for histological analysis, with Grocott's methenamine silver (GMS) for the detection of conidia and hyphae. Histopathologic scoring (HPS) was performed by a pathologist in a blinded manner. This HPS system assigned values of 0–26 (the higher the score, the greater the inflammatory changes) as described by Cimolai et al.[15]. Lungs were evaluated for inflammatory infiltrates and assigned a cumulative score in the following five categories: (1) peribronchiolar and bronchial infiltrates, (2) bronchiolar and bronchial luminal exudates, (3) perivascular infiltrates, (4) the number of neutrophils, and (5) parenchymal pneumonia.

### Bronchoalveolar lavage (BAL) for cell recovery

Airway contents were recovered by 4 rounds of filling the lungs with 0.8 ml PBS and withdrawing as much of the liquid as possible through a 20-gauge needle inserted into the trachea after cervical dissection. The bronchoalveolar lavage fluid (BALF) was centrifuged, the supernatant was removed and immediately frozen and stored at −80°C. The recovered BAL cells were resuspended in PBS and enumerated under a light microscope in the presence of trypan blue using a hemocytometer.

### Morphological leukocyte differential analysis

Cells were visually counted by standard morphological criteria in Wright-Giemsa-stained samples of BAL cell suspensions cytospun onto glass slides. For Wright-Giemsa staining, the slides were fixed for 2 min with a one-step methanol-based Wright-Giemsa stain (Sigma) followed by steps 2 and 3 of the Diff-Quik whole blood stain kit (Baxter Scientific). A total of 200 to 300 cells were counted from randomly chosen high-power microscope fields for each sample.

### Isolation and stimulation of AMs

AMs were harvested from mouse lungs with 0.8 ml ice-cold PBS (10 times) through a 20-gauge needle inserted into the trachea after cervical dissection. For stimulation assays, cells were separated from BALF by centrifugation at 300×g for 8 min at 4°C and suspended at a concentration of 2×10^6^/ml of RPMI1640 supplemented with penicillin (100 U/ml), streptomycin (100 µg/ml), and 5% heat-inactivated FBS. Aliquots of 250 µl (5×10^5^ cells) were added to 24-well plates, and allowed to adhere for 2 h at 37°C under a humidified atmosphere with 5% CO_2_. All wells were then washed three times with RPMI1640. Fresh RPMI 1640 supplemented with 10% FBS was added prior to challenge with conidia. AMs were incubated in medium alone or in the presence of viable *A. fumigatus* conidia at a microbe to cell ratio of 1∶10 (MOI = 0.1). After 6, 10, 14, and 18 hr of co-culture, total RNA from AMs was extracted, reverse-transcribed to cDNA, and stored at −20°C.

### Flow cytometry analysis

Cellular population in BALF were washed and resuspended at a concentration of 0.5×10^6^ to 1×10^6^/50 µl FA buffer (Difco) plus 0.1% NaN_3_, and Fc receptors were blocked by the addition of unlabeled anti-CD16/32(Fc blocking, eBioscience Inc.). After Fc receptor blocking, immunostaining for cell surface molecules was performed for 30 min at 4°C. Cells were washed twice with FA buffer, resuspended in 200 µl of 4% formalin (Sigma). A minimum of 10000 events were acquired on a FACSCanto flow cytometer (BD Pharmingen) using CellQuest software (BD Pharmingen). The data acquired were analyzed with FlowJo software (Tree Star, Stanford, CA). Fluorochrome-conjugated antibodies directed against the following antigens were obtained: CD45-PE, Gr-1-FITC, CD11c-APC, TLR2-FITC, TLR4-PE, (eBioscience Inc.), and Dectin-1-PE (R&D). Numbers of relevant cell types were determined by combining flow cytometry data (percentage of a given cell type).

### Gene expression

Gene expression was assessed by quantitative real-time PCR with TaqMan probes specific for mouse GAPDH (GU214026.1), TNF-α (NM_013693.2), IL-1β (NM_008361.3), IL-6 (NM_031168.1), CXCL1 (NC_000004.12), CCL3 (NC_000017.11), TLR2 (NM_011905.3), TLR4 (NM_021297.2), and dectin-1 (NM_020008.2) obtained from Applied Biosystems. RNA was isolated from lung tissue or cultured cells, and cDNA synthesis was performed using the RevertAid First Strand cDNA Synthesis Kit (Thermo Scientific) according to manufacturer's instructions. Data were expressed using the delta-delta Ct method and normalized to the housekeeping gene GAPDH. Each PCR run included a no-template control.

### Western blot analysis

Equal amounts of protein from tissue homogenates were electrophoresed through SDS-PAGE 8–18% gradient gels. The material in the gels was blotted onto a polyvinylidene difluoride (PVDF) membrane (Bio-Rad), and the membrane was blocked with 3% BSA in TBS buffer pH 7.5 for 2 hr. After washes (TBS pH 7.5, 0.02% Tween 20), immunolabeling was performed using the primary antibody at 4°C overnight. The following primary antibodies were used: anti-dectin-1 at 1∶1000, anti-TLR2 at 1∶1500, and anti-TLR4 at 1∶500 (Abcam). Then membranes were incubated with the appropriate secondary antibodies (Cell Signaling, 1∶2000) for 2 h at room temperature. Anti-GAPDH (1∶1000 Santa Cruz Biotechnology) was used as loading control. After washing, protein bands were visualized with Chemiluminescent HRP Substrate (Millipore) for 5 min at room temperature and exposed to X-ray film (Fuji Hyperfilm). Relative changes in protein expression were estimated from the mean pixel density using Quantity One software 4.6.2 (Bio-Rad), normalized to GAPHD and presented as relative density units.

### Cytokine assays

The protein levels of TNF-α, IL-1β, IL-6, CXCL1, and CCL3 in BALF were measured by commercially available cytokine-specific mouse ELISA kits (R&D) according to the manufacturers recommendations.

### Statistical analyses

Statistical analysis was performed using the Graph Pad Prism 5.0 software. Differences of survival rates were analyzed using the Log-rank and Wilcoxon tests. Parametric data were expressed as mean±SE and analyzed by t-tests. Non-parametric data were presented as median and analyzed using the Mann-Whitney test. P-values <0.05 were considered significant. All experiments were performed in triplicate.

## Results

### Vitamin D deficiency defected host resistance to *A. fumigatus*

To investigate whether vitamin D deficiency alters host resistance to *A. fumigatus*, we generated C57BL/6J mice that were nutritionally deficient in vitamin D as previously described [16]. After 8 wk on the diet, VitD- mice had 25-(OH)D3 levels of 5.1±0.25 ng/mL compared to VitD+ mice with 25-(OH)D3 levels of 82.04±1.74 ng/mL (Table 1, p<0.0001). No significant changes were observed in serum calcium and phosphorus concentrations at 8 wk on the diet (data not show). Average weight was similar in mice on VitD- and VitD+ chow over the 8 wk period (Table 1). Then, mice were infected intratracheally with viable *A. fumigatus* conidia and parameters of resistance to infection in terms of mortality, fungal growth and weight loss were evaluated.

**Table 1:** Serum concentrations of Vitamin D and weights of mice in the two dietary groups after dietary restriction.

weeks	two		four		six		eight	
	VitD+	VitD-	VitD+	VitD-	VitD+	VitD-	VitD+	VitD-
25-(OH)D3 (ng/ml)	43.4±0.67	22.3±0.89^***^	63.2±1.02	12.3±0.32^***^	75.6±1.21	8.56±0.21^***^	82.04±1.74	5.1±0.25^***^
Weight(g)	16.8±0.38	16.7±0.26	18.3±0.26	18.7±0.28	19.9±0.27	19.2±0.2	20.65±0.17	20.75±0.16

C57BL/6 mice were raised on VitD+ and VitD- diets from weaning. Every two weeks thereafter, three mice from each group were bled from the retro-orbital venous plexus and the serum concentrations of 25-(OH)D3(ng/ml) were analyzed. Values represent mean±SE (n = 3,repeated three times each). Statistical significance between the two groups of mice was evaluated using a Student's t-test p<0.001(***).

**Figure 1:**
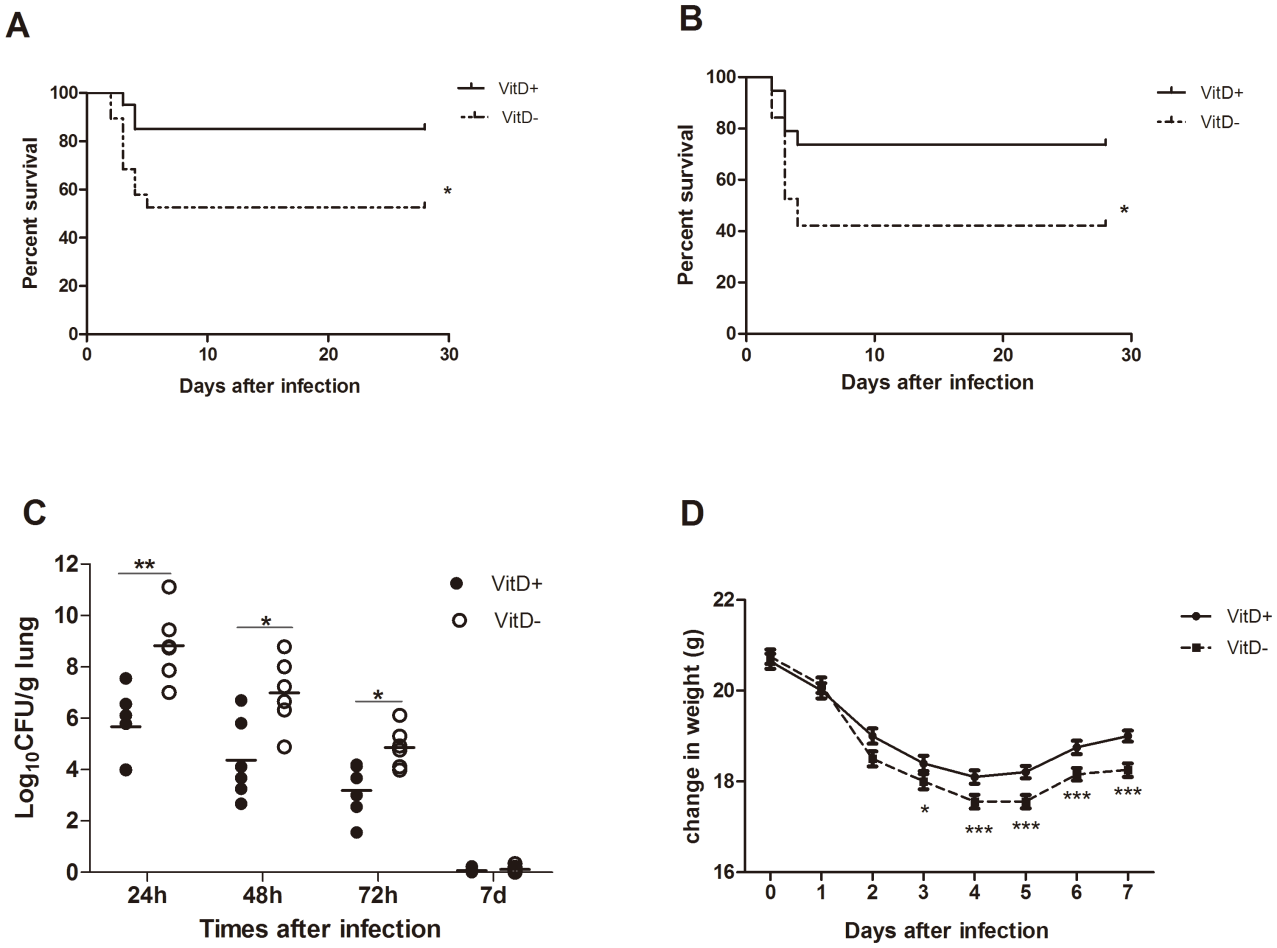
Enhanced susceptibility of VitD- mice after challenge with *A. fumigatus*. VitD+ and VitD- mice were challenged intratracheally with 5×10^7^ (A), or 7×10^7^ (B) *A. fumigatus* conidia in a volume of 50 µl. Survival was monitored for 28 days (n = 7 per group) and expressed as percentage of survival. This figure illustrates cumulative results from three independent studies per inoculum dose. The difference between survival rates was significant at p<0.05 (Log-rank test and Wilcoxon test). C. Fungal growth in the lungs of VitD+ and VitD- mice challenged intratracheally with 2×10^7^ *A. fumigatus* conidia. Mice were sacrificed at the indicated times p.i., and serial dilutions of tissue homogenates were plated for CFU determination. Results are expressed as log_10_ of CFU average values determined in triplicate of three independent experiments (three for each dilution using two animals of each group). Each data point represents one mouse. Differences in CFU between VitD+ and VitD- mice were significant at p<0.05(*) and p<0.01(**) (Mann–Whitney U test). D. Infection-induced weight changes in mice were determined daily after intratracheal challenge with 2×10^7^ *A. fumigatus* conidia. Values repersent means±SE (n = 12). p<0.001(***), p<0.01(**), p<0.05(*) (paired Student's t test).

Infection with 5×10^7^ conidia resulted in significantly higher mortality in VitD- mice than in VitD+ mice (45%; 9/20 mice versus 15%; 3/20 mice, respectively) (Fig. 1A). Increasing the inoculum to 7×10^7^ resulted in 61% mortality in VitD- mice by 4 days (Fig. 1B). Although infection with 2×10^7^ conidia resulted in no death in either group of mice in a 28-day mortality assay (data not show), vitamin D deficiency impaired pulmonary clearance of *A. fumigatus*, as determined by a significantly higher pulmonary fungal load in VitD- mice at 24, 48 and 72 hr after infection with 2×10^7^ conidia (Fig. 1C). Upon challenge with 2×10^7^ conidia, weight loss was more pronounced in VitD- mice, and weight gain in VitD- mice was far less than in VitD+ mice (Fig. 1D). All these date suggested that vitamin D deficiency resulted in a defective host pulmonary resistance to *A. fumigatus*.

### Aggravated and sustained inflammatory response in *A. fumigatus*-challenged VitD- mice

#### Histological evidence of aggravated and sustained inflammatory response in *A. fumigatus*-challenged VitD- mice

**Figure 2:**
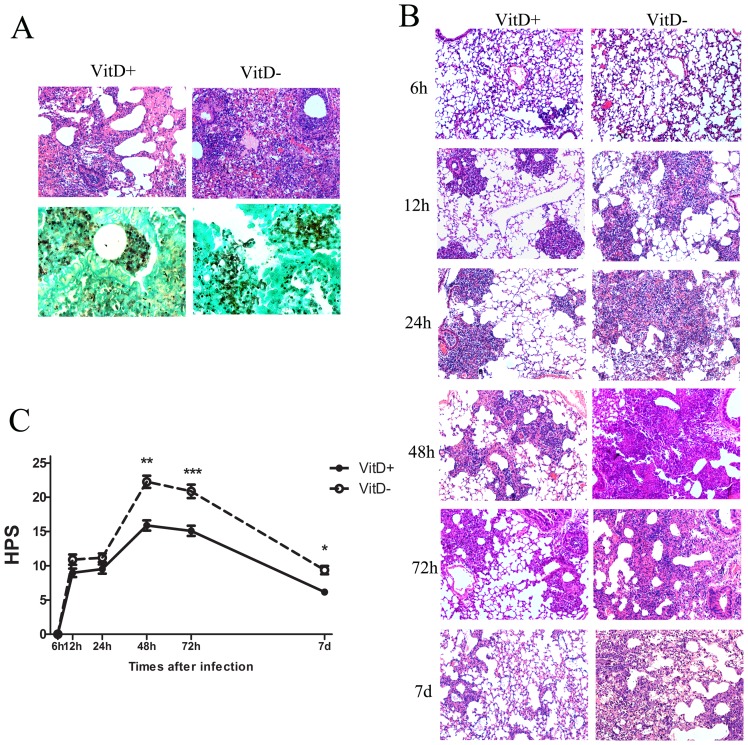
Histological evidence of aggravated and sustained inflammation in VitD- mice challenged with *A. fumigatus* conidia. A. Representative lung sections from VitD+ and VitD- mice three days post intratracheal challenge with 5×10^7^ *A. fumigatus* conidia. Sections were stained with HE (upper, magnification 100x) for analysis of inflammation, and with GMS (lower, magnification 400x) for the detection of conidia and hyphae. B. Representative HE-stained lung sections (magnification 100x) from VitD- and VitD+ mice are shown at the indicated times after intratracheal challenge with 2×10^7^ *A. fumigatus* conidia. C. HPS of serial HE-stained lung sections between 6 h and 7 d after intratracheal challenge with 2×10^7^ *A. fumigatus* conidia. HPS is presented as mean±SE. Data are representative of three independent experiments (n = 3/group). Statistical analysis was performed by a Mann-Whitney rank sum test. p<0.001(***), p<0.01(**), p<0.05(*).

HE staining of lung tissue sections revealed the presence of inflammatory cells, mainly neutrophils, centered on bronchi/bronchioles and blood vessels with signs of peribronchial necrosis in both groups of mice (Fig. 2A, upper, 72 hr after challenge with 5×10^7^ *A. fumigatus* conidia). The inflammatory lesions in VitD- mice were more severe and diffused, with peribronchiolar edema, consolidation of the inflammatory response, and disappearance of alveolar structure (Fig. 2A). GMS staining of lung tissue sections revealed *A. fumigatus* germinating conidia and septate-branching hyphae in VitD- mice as opposed to the relatively fewer germinating conidia and no hyphae in VitD+ mice (Fig. 2A, below).

Infection with 2×10^7^ conidia resulted in no death in either group of mice. So lung histopathology was analyzed sequentially (Fig. 2B) and lung inflammation was quantified by HPS (Fig. 2C) after challenge with this amount of inocula. By 6 hr, lung sections remained without significant histological changes. By 12 hr and 24 hr, early scattered areas of inflammation were observed, and were more apparent in VitD- mice. By 48 hr and 72 hr, differences were notable, with more profound and intense inflammatory response in VitD- mice. By 7 d, the inflammation in the VitD+ lung sections apparently subsided, while inflammatory cell infiltration and fibrinous exudation were still noted in VitD- mice. The HPS first increased at 12 hr, peaked at 48 hr, and declined thereafter, and it was significantly higher in VitD- mice than in VitD+ mice at 48 hr, 72 hr and 7 d p.i. (Fig. 2C). Thus, histopathologic analysis revealed more severe inflammation with delayed resolution in VitD- mice compared to their VitD-sufficient counterparts, although germinating conidia and hyphae could hardly be found in both groups of lungs at all the indicated times p.i. (no shown).

**Increased infiltration of inflammatory cells in the lungs of *A. fumigatus*-challenged VitD- mice**. We investigated cell numbers and composition in BALF after *A. fumigatus* challenge. Mice were challenged with 2×10^7^ *A. fumigatus* conidia, and the amounts of cells in BALF were enumerated using a hemocytometer. BAL Cells were identified by morphology after staining with Wright-Giemsa stain, subsequently sorted by flow cytometry.

**Figure 3:**
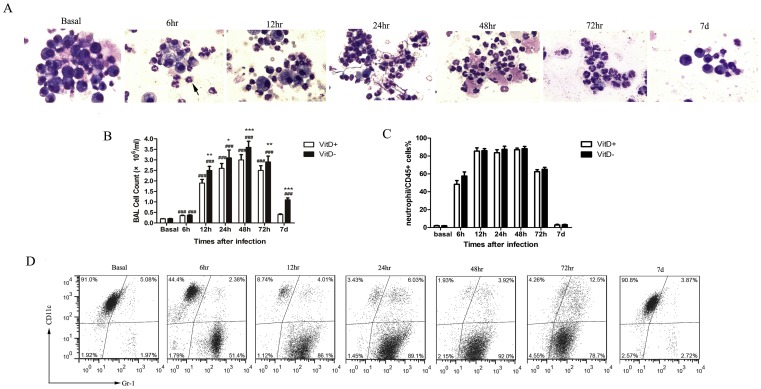
Increased infiltration of inflammatory cells in the lungs of *A. fumigatus*-challenged VitD- mice. A. BAL cells were stained with Wright-Giemsa stain and examined under a light microscope after intratracheal challenge with 2×10^7^ *A. fumigatus* conidia (magnification 400x). B. Total cell counts in BALF were enumerated on a hemacytometer of individual mice after intratracheal challenge with 2×10^7^ conidia. Data are representative of three independent experiments with n = 3 per group (mean±SE) and analyzed by paired Student's t test. p<0.001(###), for comparison of *A. fumigatus*-challenged VitD+/VitD- mice versus baseline levels; p<0.001(***), p<0.01(**), p<0.05(*), for comparison of VitD+ with VitD- mice. C. BAL cells were analyzed by flow cytometry after intratracheal challenge with 2×10^7^ conidia. Lavage cells were gated by CD45^+^; the resulting populations were further identified on the basis of CD11c and Gr-1expression and sorted as shown by the density plots. Within the CD45^+^ population, the major cell types were macrophages (CD11c^+^Gr-1^+/−^) at baseline, neutrophils (CD11c^−^Gr-1^+^) became the major cell types 6–72 hr post infection. Flow cytometry was performed to quantify the percentage of cell types in CD45^+^ cells. D. Flow cytometry was performed to quantify the percentage of neutrophils in CD45^+^ cells in BALF after intratracheal challenge with 2×10^7^ conidia. Neutrophils (CD11c^−^Gr-1^+^) were gated from the CD45^+^ cells based on Gr-1 and CD11c expression profiles. Data are representative of three independent experiments with n = 3 per group (mean±SE), and analyzed by paired Student's t test.

At baseline (no exposures), the BALF consisted predominantly of AMs (Fig. 3A). No differences were observed in the basal counts of BAL cells from VitD+ and VitD- mice (Fig. 3B). *A. fumigatus* challenge resulted in significant increase in the number of inflammatory cells in BALF (Fig. 3B), which was accounted for by an increase in neutrophils (Fig. 3A, arrow indicated). BAL cells were dominated by neutrophils from 12 hr to 72 hr p.i., eosinophils and lymphocytes could hardly be found in the BAL smears (Fig. 3A). The total number of inflammatory cells in BALF peaked at 48 hr p.i., and was greater in VitD- mice compared with VitD+ mice at all the indicated time points p.i., except 6 hr (Fig. 3B). We found no differences in the proportion of neutrophils in the BAL smears between VitD+ and VitD- mice at any of the indicated times p.i. (data not shown).

Based upon the morphological observation of BAL smears and the staining strategies previously reported [17], [18], a panel of cell surface antibodies against CD45, CD11c, and Gr-1 was used for differential flow cytometry staining of cells obtained by BAL. CD45^+^ BAL cells fell within one of four distinct populations on a Gr-1/CD11c plot. The Gr-1^−^CD11c^−^ population in the BALF consisted of lymphocytes, Gr-1^+^CD11c^−^ of neutrophils, Gr-1^lo/−^CD11c^hi^ and Gr-1^+^CD11c^hi^ of macrophages [17], [19]. At baseline (no exposures), within the CD45^+^ population of cells, the major cell types were macrophages (Gr-1^lo/−^CD11c^hi^ and Gr-1^+^CD11c^hi^), accounting for 95% of CD45^+^ cells (Fig. 3D). Following inoculation with *A. fumigatus*, there was a rapid and substantial increase in the numbers of neutrophils (Gr-1^+^CD11c^−^), which were dramatically increased from <2% of total BAL leukocytes at baseline to 50% of total BAL leukocytes by 6 hr, accounted for >85% of total BAL leukocytes at 24 hr and 48 hr p.i. (Fig. 3D). No differences were observed in the percentage of neutrophils in BAL leukocytes from VitD+ and VitD- mice at any time point p.i. (Fig. 3C). We also did not observe any differences in the proportion of macrophages in BAL leukocytes (data not show). Lymphocytes (Gr-1^−^CD11c^−^) were no more than 5% of total BAL leukocytes at any of the indicated times. The numbers of cell populations returned to the baseline at 7 days p.i. (Fig. 3C, D).

Altogether, these data demonstrated that *A. fumigatus* challenge induced infiltration of inflammatory cells, dominated by neutrophils. Vitamin D deficiency did not affect the proportion of neutrophils in BAL leukocytes in response to *A. fumigatus* challenge.

**Figure 4:**
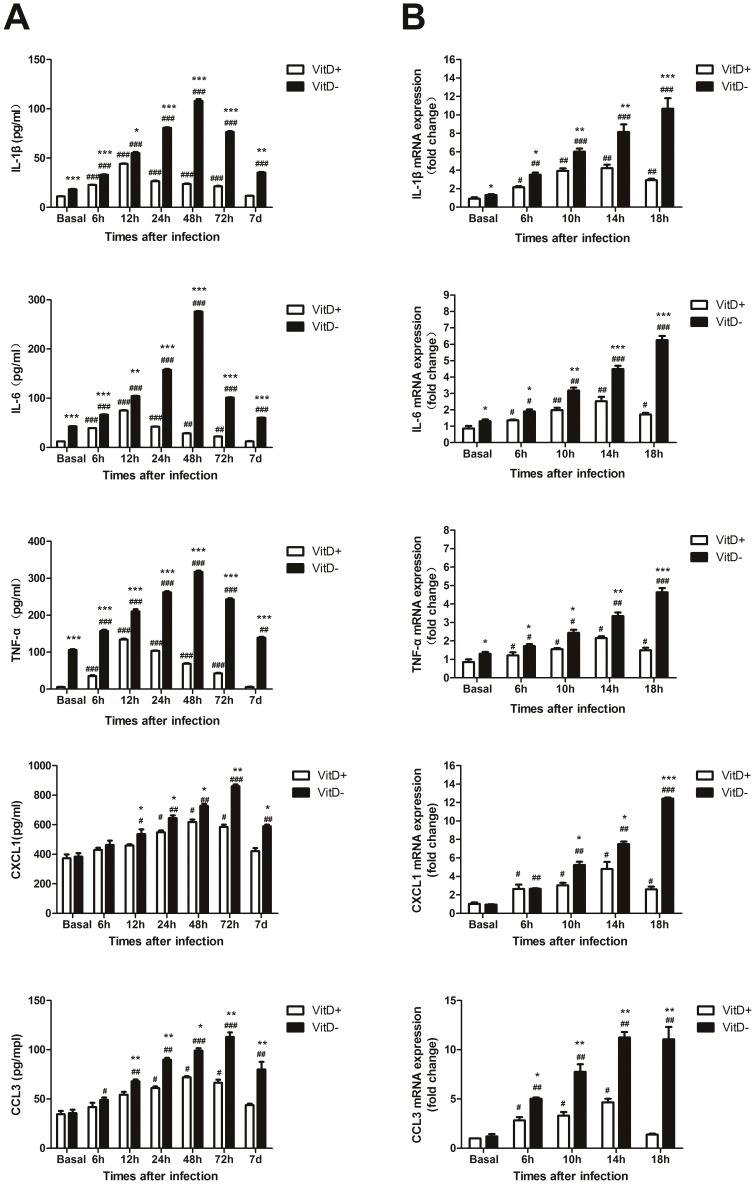
Enhanced pro-inflammatory cytokine and chemokine production in the lungs of *A. fumigatus*-challenged VitD- mice. A. Mice were infected intratracheally with 2×10^7^ *A. fumigatus* conidia. At specific times p.i. BALF collected from mice was analyzed for cytokine and chemokine content by ELISA. Data are representative of three independent experiments with n = 5 per group (mean±SE). p<0.001(###), p<0.01(##), p<0.05(#), for comparison of *A. fumigatus*-challenged VitD+/VitD- mice versus baseline levels; p<0.001(***), p<0.01(**), p<0.05(*), for comparison of VitD+ with VitD- mice using paired Student's T-test. B. AMs isolated from naïve mouse lungs by BAL, were incubated in medium alone or in the presence of viable *A. fumigatus* conidia (MOI = 0.1) *in vitro*. Total RNA from AMs was extracted at indicated times. Transcript abundance was measured by real-time RT-PCR. Representative data from three independent experiments with n = 3 per group are shown. P<0.001(###), P<0.01(##), P<0.05(#), for comparison of *A. fumigatus*-challenged VitD+/VitD- AMs versus basal levels; P<0.001(***), P<0.01(**), P<0.05(*), for comparison of VitD+ and VitD- AMs using paired Student's T-test.

**Enhanced pro-inflammatory cytokine and chemokine production in the lungs of *A. fumigatus*-challenged VitD- mice**. We next investigated the kinetic production of pro-inflammatory cytokine and chemokine in lung to better understand the inflammatory responses occurring after exposure. Vitamin D deficiency significantly enhanced the basal expression of IL-1β, IL-6, and TNF-α in the lungs of mice, while did not influence the basal expression of CXCL1 and CCL3 (Fig. 4A). *A. fumigatus* infection elicited a robust inflammatory response in lung, characterized by the increased production of IL-1β, IL-6, and TNF-α. This response was more profound in the lungs of VitD- mice at all the indicated times p.i. (Fig. 4A). Upon infection, the levels of these cytokines were elevated from 6 hr on; in VitD+ mice they peaked at 12 hr, returned to baseline at 7 d; in contrast in VitD- mice, they peaked at 48 hr, and were still significantly elevated over baseline levels at 7 d p.i. VitD- mice had significantly higher amounts of CXCL1 and CCL3 versus VitD+ mice at 12 to72 hr, and 7 d p.i. (Fig. 4A). In VitD+ mice they peaked at 48 hr, returned to baseline at 7 d p.i.; while in VitD- mice they peaked at 72 hr, and were still significantly higher than baseline at 7 d p.i.

Because AMs are a critical source for pro-inflammatory cytokine and chemokine production in response to *A. fumigatus* [20], we isolated AMs from naïve VitD+ and VitD-mice and assessed the kinetic pro-inflammatory cytokine and chemokine production in response to live *A. fumigatus* conidia *in vitro*. Results showed that compared with VitD+ AMs, VitD- AMs had significantly higher basal levels of IL-1β, IL-6, and TNF-α (Fig. 4B). In response to *A. fumigatus* conidia, VitD- AMs still had higher levels of them versus VitD+ AMs at all the indicated times. No differences in the basal levels of CXCL1 and CCL3 expression were observed between VitD+ and VitD- AMs (Fig. 4B). In response to *A. fumigatus* conidia, VitD- AMs had significantly higher amounts of CXCL1 and CCL3 versus VitD+ AMs at 10 hr, 14 hr and 18 hr. Furthermore, the up-regulation of cytokines and chemokines in VitD+ AMs peaked at 14 hr, decreased rapidly thereafter; while in VitD- AMs their expression showed a continuously rising trend for 18 hr (Fig. 4B).

These results suggested that vitamin D deficiency enhanced the basal levels of pro-inflammatory cytokines in the lungs of mice. Upon *A. fumigatus* infection, vitamin D deficiency led to enhanced and sustained expression of pro-inflammatory cytokines and chemokines both *in vivo* and *in vitro*.

**Figure 5:**
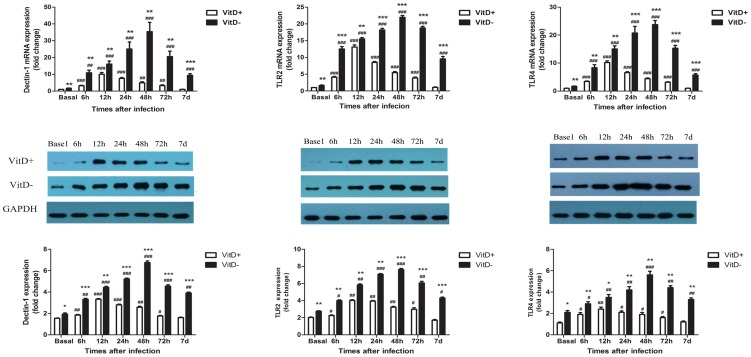
Enhanced and prolonged up-regulation of PRRs in the lungs of *A. fumigatus* challenged VitD- mice. Mice were infected intratracheally with 2×10^7^ *A. fumigatus* conidia. At specific time p.i., mice were sacrificed and lungs were excised, and total RNA and protein was extracted. Expression of dectin-1, TLR2 and TLR4 at the mRNA level was quantified by real-time RT-PCR, and protein levels were quantified by Western blotting (shown in middle panel). Representative data from three independent experiments are shown with n = 3 per group (mean±SE). p<0.001(###), p<0.01(##), p<0.05(#), for comparison of *A. fumigatus*-challenged VitD+/VitD- versus baseline levels; p<0.001(***), p<0.01(**), p<0.05(*), for comparison of VitD+ and VitD- mice using paired Student's T-test.

**Enhanced up-regulation of pattern recognition receptors (PRRs) both at baseline and post A. fumigatus challeng in the lungs of VitD- mice**. The initial sensing of fungal infection is mediated by PRRs, such as TLRs and lectin receptors, which trigger intracellular signaling cascades, leading to transcriptional expression of inflammatory mediators [21], [22]. TLR2, TLR4 and _β-glucan receptor dectin-1 are key PRRs known to be involved in *A. fumigatus* recognition [21]. Hence, we investigated whether vitamin D deficiency modulates the expression of those PRRs in the lungs of mice (Fig. 5). Results showed that vitamin D deficiency increased pulmonary TLR2, TLR4 and dectin-1 expression at baseline both at mRNA and protein levels. Upon *A. fumigatus* challenge, these PRRs expression were up-regulated significantly, while VitD- mice still had higher mRNA and protein levels of these PRRs than VitD+ mice at all the indicated times. In VitD+ mice, these PRRs peaked at 12 hr, returned to baseline levels at 7 d p.i. While, in VitD- mice, they peaked at 48 hr, declined slowly thereafter, and were still much higher than basal levels at 7 d p.i.

**Figure 6:**
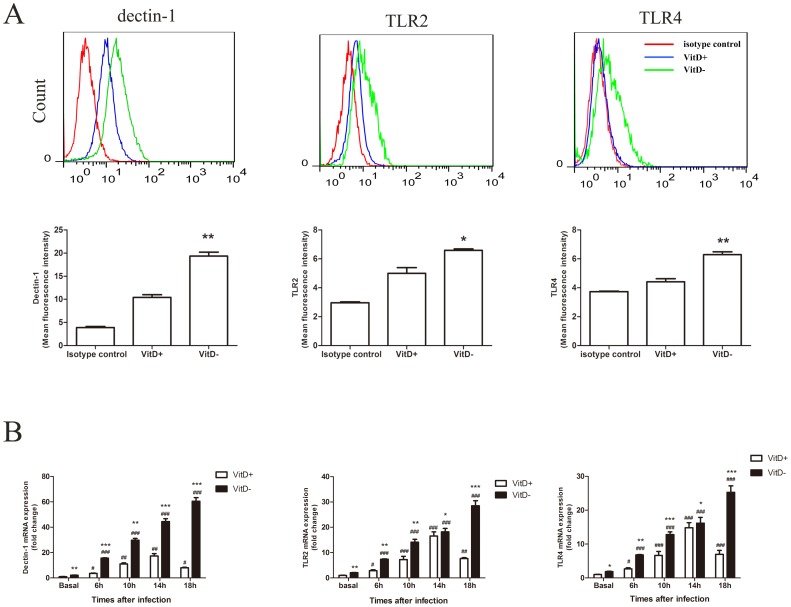
Enhanced and prolonged up-regulation of PRRs on *A. fumigatus*-stimulated VitD- AMs. A. Expression of surface TLR2, TLR4 and dectin-1 on AMs isolated from VitD- or VitD+ mice. AMs were isolated from mouse lungs by BAL, and stained for CD11c, dectin-1, TLR2 or TLR4 and then analyzed using flow cytometry. The data show the expression of dectin-1, TLR2 and TLR4 on CD11c-positive cells (red line, isotype control; blue line, VitD+ AMs; green line, VitD- AMs). The values shown in the flow cytometry profiles are the mean fluorescence intensity (MFI) indices. Data are representative of three independent experiments with n = 3/group. p<0.01(**), p<0.05(*) using paired Student's T-test. B. mRNA expression of TLR2, TLR4 and dectin-1 in AMs after co-cultured with live *A. fumigatus* conidia *in vitro*. AMs were isolated from naïve mouse lungs by BAL, incubated in medium alone or in the presence of viable *A. fumigatus* conidia (MOI = 0.1). Total RNA from AMs was extracted at indicated times. Transcript abundance was measured by real-time RT-PCR. Representative data from three independent experiments with n = 3 per group are shown. P<0.001(###), P<0.01(##), P<0.05(#), for comparison of A. f.-challenged VitD+/VitD- versus baseline levels; P<0.001(***), P<0.01(**), P<0.05(*), for comparison of VitD+ and VitD- using paired Student's T-test.

Because AMs are of the main cells that express PRRs and recognize microorganism [20]. We investigated whether vitamin D deficiency affects the expression of TLR2, TLR4, and dectin-1 on AMs at baseline by flow cytometry. We found that the expression of these PRRs on AMs isolated from naïve VitD- mice was up-regulated significantly compared to AMs from naïve VitD+ mice (Fig. 6A). We further investigated the expression of these PRRs on AMs in response to *A. fumigatus in vitro*. AMs were isolated and co-cultured with viable *A. fumigatus* conidia. Gene expression of TLR2, TLR4, and dectin-1 was analyzed by q-PCR. Results showed that *A. fumigatus* challenge significantly increased the expression of these PRRs *in vitro* (Fig. 6B). Similar to what was found in vivo, increased expression of these PRRs was more pronounced in VitD- AMs compared with VitD+ AMs (Fig.6B). The up-regulation of PRR genes in VitD+ AMs peaked at 14 hr p.i. and decreased rapidly thereafter, while PRR gene expression showed a continuously rising trend for 18 hr in VitD- AMs (Fig. 6B).

Thus, vitamin D deficiency caused enhanced up-regulation of TLR2, TLR4 and dectin-1 in the lungs of mice both at baseline and post *A. fumigatus* challenge.

## Discussion

Our results showed that VitD- mice demonstrated higher mortality after lung challenge with *A. fumigatus* coupled with impaired early clearance of the organism from the lungs. VitD- mice demonstrated aggravated and prolonged histological evidence of lung inflammation as well as enhanced BAL cell counts, dominated by neutrophils after *A. fumigatus* inoculation. Increased basal levels of pro-inflammatory cytokines in the lungs and AMs from naïve VitD- mice were observed. Upon *A. fumigatus* exposure, vitamin D deficiency led to enhanced and sustained expression of pro-inflammatory cytokines and chemokines both *in vivo* and *in vitro*. Up-regulation of key PRRs involved in *A. fumigatus* recognition was observed in the lungs and AMs from VitD- mice both at baseline and after *A. fumigatus* exposure.

Our survival experiments were performed with inocula of 5–7×10^7^conidia, based on published reports using inocula at this level when assessing mortality and fungal clearance in animals that were not dually immunosuppressed with cyclophosphamide and cortisone [23], [24]. Our experiments were not conducted in the presence of immunosuppressive drugs (cyclophosphamide, cortisone acetate, etc.) or during transient neutropenia (i.e., Ab-mediated depletion of neutrophils). Our position was that induction of immunosuppression before infection might exaggerate or underestimate the role of vitamin D whose importance in lung clearance of *A. fumigatus* in immunocompetent mice was clearly supported by the data of our experiments.

An important finding of this study was that the expression of pro-inflammatory cytokines (TNF-α, IL-1β and IL-6) and key PRRs involved in *A. fumigatus* recognition (TLR2, TLR4, and dectin-1) was significantly elevated in the lungs and AMs of VitD- mice versus VitD+ mice already at baseline. Microbial infection causes cytokine-associated inflammation to remove pathogens. The initial sensing of infection is mediated by innate PRRs. The intracellular signaling cascades triggered by PRRs recognition lead to transcriptional expression of inflammatory mediators that coordinate the elimination of pathogens and infected cells [22]. Studies have confirmed that naïve mice lacking dectin-1 or TLRs signaling were more sensitive to intratracheal challenge with *A. fumigatus* than control mice, and demonstrated impaired pro-inflammatory cytokines production and insufficient lung neutrophil recruitment [24], [25]. Mice lacking TNF-α, or CCR1 (receptor for CCL3), were more susceptible to *A. fumigatus* lung infection, also through impaired neutrophil recruitment [26], [27]. We thus expected that the increased basal levels of these PRRs and pro-inflammatory cytokines in the lungs of VitD- mice, which led to enhanced release of pro-inflammatory cytokines and chemokines after lung challenge with *A. fumigatus*, accompanied by more neutrophil infiltration in the lungs in our model, might increase host defense to *A. fumigatus*. However, the data in our study showed that up-regulation of them did not improve host resistance to *A. fumigatus*.

Excessive or uncontrolled release of pro-inflammatory cytokines can be fatal, which causes immunodeficiency, septic shock, or induction of autoimmunity, and may eventually impair disease eradication [22], [28], [29]. Influenza A virus and some Gram-negative bacteria trigger life-threatening “cytokine storms” in the host resulting in significant pathology and ultimately death [28], [30]. It has been proposed that in invasive fungal infections, disease pathology may also be attributable to an aggravated or dysregulated host inflammatory response that results in extensive tissue damage [14], [29], [31]. In mice with chronic granulomatous disease, the intrinsic, genetically determined failure to control inflammation after exposure to sterile fungal components determines the animals' inability to resolve an infection with *A. fumigatus* [32]. Chronic disseminated candidiasis (CDC) is typically observed during neutrophil recovery in patients with acute leukemia, and the efficacy of corticosteroid therapy supports the pathophysiological hypothesis that CDC is a fungus-related immune reconstitution inflammatory syndrome [33]. The active form of vitamin D has been proved to inhibit *M. tuberculosis*, virus and bacteria through the induction of the cathelicidin antimicrobial peptide and β-defensins[34], [35], or autophagy[36], while decreasing the inflammatory response to microbial infections[37], [38], [39], [40], [41]. Upon *in vitro* LPS, *C. albicans*, and *M. tuberculosis* stimulation, the active form of vitamin D has been observed to inhibit the production of TNF-α, IL-1β, and IL-6 by monocytes directly, or through down-regulating the expression of TLR2, TLR4 and dectin-1 on monocytes [39], [40], [41], [42], [43]. An increased serum vitamin D3 level in summer was associated with a significant drop in pro-inflammatory cytokines production by human monocytes *in vitro*, when compared to cytokine production in winter [44]. In addition, the reduced expression of TLR2 and TLR4 on human monocytes was associated with elevated vitamin D levels during summer, and the reduction of TLR2 expression during summer was accompanied by a corresponding drop in TNF-α production by human monocytes *in vitro* [44]. The active vitamin D can be produced locally within the lungs. Vitamin D deficiency resulted in decreased synthesis of active vitamin D in lung. Therefore, it is possible that lack of vitamin D increased the basal levels of pro-inflammatory cytokines and PRRs in the lungs of mice, which subsequently led to a hyper-inflammatory response to *A. fumigatus* in lung and impaired host resistance to *A. fumigatus*.

Neutrophils are essential in initiation and execution of the acute inflammatory response in fungal infection [45]. 1 alpha, 25-Dihydroxyvitamin D3 was demonstrated to inhibit neutrophil motivation by decreasing ICAM-1 and ELAM-1 expressions on pulmonary microvascular endothelial cells [46], and inhibited neutrophil recruitment in lung in animal models of acute lung injury[47], [48]. In addition, the active form of vitamin D was observed to suppress IL-8 production in Pseudomonas-stimulated neutrophils and have benefit for resisting persistent *P. aeruginosa* lower respiratory tract infection [49]. Thus, vitamin D deficiency might lead to more neutrophils infiltration in lung in response to *A. fumigatus* challenge, which aggravated pulmonary inflammatory response.

Moreover, our experiments were conducted in immunocompetent mice. In this case, the increased fungal burden in the lungs of VitD- mice might further enhance the PRRs signal, and exacerbated the values of inflammatory mediators, which aggravated the inflammation in the lungs, and eventually led to a poor prognosis.

Human diseases caused by *A. fumigatus* have been considered under three categories: invasive infections; infections caused by the colonization of mucosal surfaces without invasion into tissue; and hypersensitivity diseases. The key determinant of the pathogenicity of Aspergillus species, and the reason for the diversity of host outcomes, is hypothesized to be the nature of the immune response of the host [50], and the diseases may be conceptualized as points along a spectrum of abnormal immune responses of the host. Vitamin D may have a role in inhibiting *A. fumigatus* infections, while modulating and limiting any unnecessary inflammatory response generated by the host. In recent years, vitamin D deficiency in humans has received significant attention [7], [51], [52]. It has been shown to play a potent role in the susceptibility to respiratory infectious diseases [53], [54]. A still unresolved question arising from ongoing laboratory and clinical research is the serum vitamin D concentrations needed to elicit an optimal immune response [55]. Our study indicates that vitamin D deficiency might lead to an aberrant immune response to *A. fumigatus*.

In conclusion, we have identified an important role of vitamin D for host defense against *A. fumigatus* in an immunocompetent host. By modifying the pro-inflammatory cytokine responses, and influencing on the major PRRs involved in *A. fumigatus* recognition, vitamin D deficiency causes an aggravated and prolonged inflammatory response in challenge with *A. fumigatus* infection. We therefore feel these studies lay the foundation for further examination of vitamin D status in individuals who are at risk for Invasive Pulmonary Aspergillosis (IPA), which may provide valuable insight into the role of vitamin D in susceptibility to IPA in immunosuppressed patients.
